# Recent advances in metal–organic framework as nanocarriers for pesticide delivery

**DOI:** 10.3389/fpls.2026.1776360

**Published:** 2026-03-18

**Authors:** Fengping Yang, Lei Han, You Liang, Zhongyang Huo

**Affiliations:** 1 College of Bioscience and Biotechnology, Yangzhou University, Yangzhou, China; 2 Co-Innovation Center for Modern Production Technology of Grain Crop/Jiangsu Key Laboratory of Crop Genetics and Physiology, Yangzhou University, Yangzhou, China

**Keywords:** crop protection, metal-organic frameworks, pesticide delivery, stimuli-responsive release, sustainable agriculture

## Abstract

The excessive use of conventional agrochemicals has led to severe environmental contamination, low utilization efficiency, and increasing concerns regarding ecological safety and sustainability. In recent years, metal–organic frameworks (MOFs) have emerged as a versatile class of porous materials for advanced agrochemical delivery owing to their high surface area, tunable pore structure, and modular chemical design. This review systematically summarizes the recent progress in MOF-based agrochemical delivery systems, with particular emphasis on their synthesis evolution, structural characteristics, functional modification strategies, and environmentally responsive release behaviors. MOF-based nanoformulations are categorized according to their agricultural applications, including insecticides, fungicides, herbicides, and plant growth regulators. Representative systems are discussed to elucidate structure–performance relationships in terms of loading capacity, release kinetics, biological efficacy, and field-relevant performance. In addition, the multifunctional roles of MOFs as both delivery carriers and potential sources of beneficial macro- and micronutrients are highlighted, demonstrating their capacity to integrate crop protection with plant health management. Furthermore, critical challenges associated with MOF-based agrochemical systems are analyzed, including synthesis sustainability, environmental fate, biosafety, economic feasibility, and regulatory acceptance. Application-specific ecological risks, long-term behavior in soil–plant–water systems, and the need for standardized evaluation frameworks are discussed in detail. Finally, future perspectives are proposed to guide the rational design and practical implementation of MOF-enabled agrochemical technologies, emphasizing green synthesis, cost reduction, scalability, and farmer adoption. Overall, this review provides a comprehensive overview of MOF-based agrochemical delivery systems and offers insights into their potential role in advancing sustainable and intelligent modern agriculture.

## 1 Introduction

According to the United Nations Population Fund (UNFPA), the global population is projected to reach 9.7 billion by 2050 and exceed 10.4 billion by 2100 (Washington and Kopnina, 2022). To meet the growing demand for food production, pesticides (including fungicides, insecticides, and herbicides) have been extensively applied to control plant diseases, insect pests, and weeds (Angon et al., 2023; Tang et al., 2025; Zhu Z, et al., 2025). According to Food and Agriculture Organization (FAO) statistics, global pesticide consumption reached approximately 3.7 million tons in 2022, representing nearly a 50% increase compared with 1990 and reflecting a growing dependence on chemical inputs in modern agriculture (Hamed and Hidayah, 2025; Leskovac and Petrović, 2023). However, the effective utilization efficiency of conventional pesticide formulations remains extremely low, as nearly 90% of the applied active ingredients are lost to the environment through volatilization, runoff, leaching, or photodegradation rather than reaching their intended biological targets (Anjaneyulu et al., 2024; Vishnu et al., 2024; Zhou et al., 2024). This excessive loss not only leads to substantial waste of resources but also intensifies ecological risks, including soil degradation, biodiversity decline, and the accelerated evolution of pesticide resistance in pest and pathogen populations (Rodrigues et al., 2024; Zambela et al., 2025; Zhou et al., 2025). These limitations underscore the urgent need for innovative, sustainable, and high-efficiency pesticidal technologies capable of improving delivery accuracy while mitigating environmental burdens.

In recent years, controlled-release pesticide formulations have been developed as promising alternatives to conventional pesticide technologies. Polymer coatings, microencapsulation, and matrix embeddings allow temporal and spatial modulation of pesticide release, enhancing bioefficacy and prolonging in-field persistence while reducing application frequency (Hellmann et al., 2024; Lobel et al., 2024; Verma et al., 2025; Wang et al., 2024a; Xu et al., 2024, Xu et al., 2026; Zhang et al., 2024, Zhang et al., 2025). However, most existing controlled-release systems rely on passive release mechanisms, such as diffusion or carrier degradation, which lack responsiveness to the dynamic agricultural environment and often lead to undesirable burst release or delayed activation under fluctuating field conditions (Arunachalam et al., 2025; Brunelle et al., 2024; Islam et al., 2025; Iyarin Thanka Mahil et al., 2025; Ma et al., 2025; Nasif et al., 2024; Wang et al., 2025, Wang et al., 2022; Zeng and Motola, 2025). With the rise of precision agriculture and the growing emphasis on green plant protection, research has increasingly shifted toward environment-responsive (stimuli-responsive) nano-pesticide delivery systems, regarded as next-generation platforms for smart crop protection. These systems are engineered with functional groups or structures that respond to specific environmental cues, such as pH, temperature, enzyme activity, redox conditions, or light irradiation, thereby enabling site-specific and on-demand pesticide release (Li et al., 2024; Liang et al., 2025, Liang et al., 2022a). A growing body of evidence indicates that pathogen infection and pest feeding can induce localized changes in the plant or soil microenvironment, including acidification, secretion of specific enzymes (e.g., cellulase, pectinase, protease), and altered redox states (Li et al., 2025; Wang et al., 2025, Wang et al., 2024c). Thus, the development of environmentally responsive nano-carriers has emerged as a promising strategy for achieving precise pest control while reducing chemical inputs.

A wide range of nanocarriers, including organic “soft” nanoparticles, inorganic “hard” nanoparticles, nanoclays, and carbon-based materials, have been explored to improve the dispersibility, stability, and delivery efficiency of pesticide active ingredients (Bueno et al., 2022; Dhiman et al., 2022; Pal et al., 2023; Sarlak et al., 2014). However, these systems still suffer from several inherent drawbacks, such as limited structural tunability, long-term instability, uncontrolled burst release, and potential biotoxicity or persistence, which constrain their reliability and environmental compatibility under realistic field conditions (Gamoń et al., 2023; Granetto et al., 2022; Vemula and Reddy, 2023; Wang et al., 2023; Wei et al., 2024). Metal-organic frameworks (MOFs) have recently emerged as a promising solution to these limitations (Rojas et al., 2022). Constructed via the coordination-driven self-assembly of metal ions or clusters with organic ligands, MOFs possess highly ordered three-dimensional porous architectures, ultrahigh surface areas, abundant functionalizable sites, and excellent thermal stability and biocompatibility (Yusuf et al., 2022). Their modular tunability, achieved through the variation of metal nodes and organic linkers, enables rational engineering of pore environments and surface properties, allowing MOFs to function both as high-capacity carriers and as selective release mediators (Nasri et al., 2025). Importantly, the coordination bonds, pore accessibility, and surface chemistry of many MOFs are intrinsically sensitive to external stimuli, enabling frameworks to undergo reversible or partially reversible structural adjustments under changes in pH, temperature, light, redox conditions, or enzymatic activity. These structural responses directly influence guest–host interactions, molecular diffusion, and spatial confinement, providing built-in mechanisms for environmentally responsive, directional pesticide release (Fang et al., 2024; Liang et al., 2022a, Liang et al., 2022b). Furthermore, a variety of MOFs are environmentally degradable, and their breakdown products, such as low-toxicity metal ions or small organic ligands, pose minimal ecological risk and can even act as supplemental micronutrients for crops (Rawat et al., 2025).

Recent advances in MOF synthesis, including hydrothermal routes, one-pot assembly, vapor diffusion, and coordination modulation, have enabled fine control over particle size, crystallinity, defect density, and pore architecture, laying the foundation for rational design of MOF-based agricultural nanocarriers (Daliran et al., 2024; Liu et al., 2024; Perveen et al., 2025). Further functionalization of MOFs using polymer gatekeepers, inorganic shell deposition, or supramolecular valves can effectively seal their pores and prevent the premature leakage of pesticide active ingredients (Chen et al., 2025a; Gao et al., 2021a; Yang et al., 2021). These modifications enhance the stability, improve utilization efficiency, and reduce environmental losses of the loaded pesticides during field application (Zhang et al., 2025).

**Figure 1:**
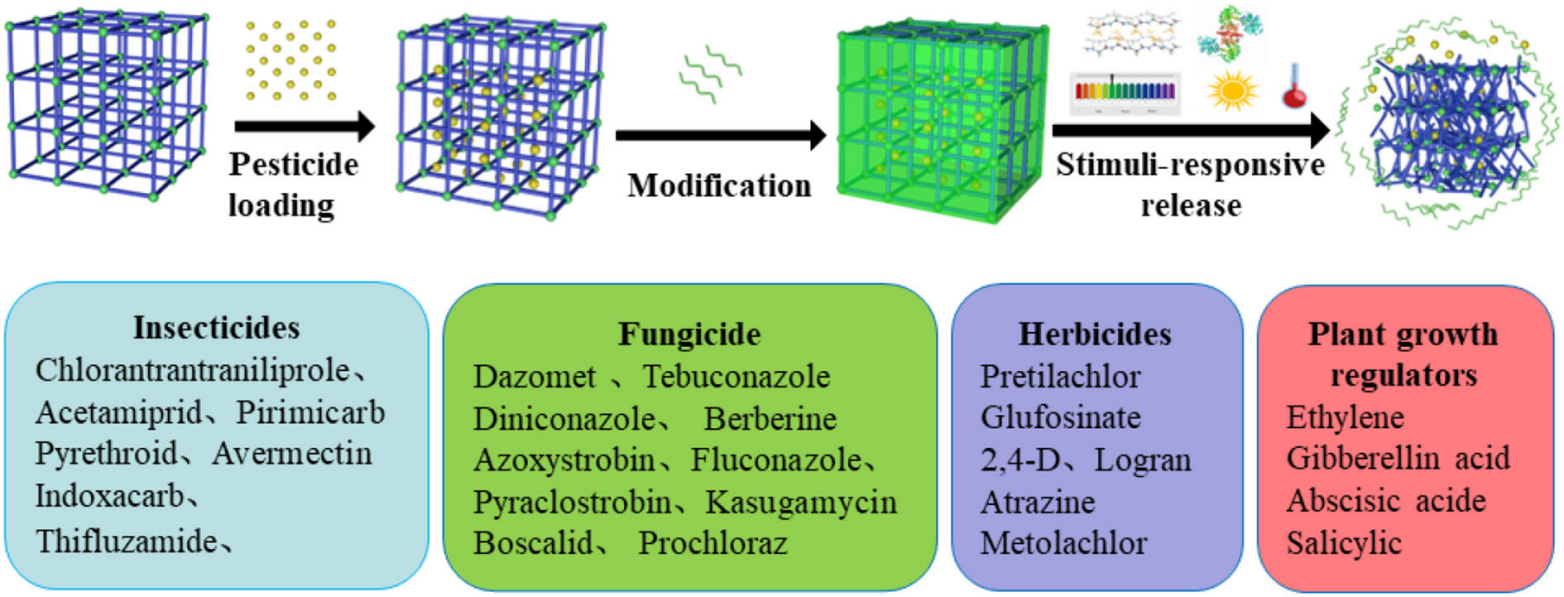
Application of MOFs in Pesticide Controlled Release.

Although MOFs have been widely studied in areas such as gas storage, catalysis, and biomedicine, their rapidly expanding applications in agriculture, especially as pesticide carriers, have not yet been comprehensively reviewed (Felix Sahayaraj et al., 2023; Liu et al., 2024; Salehipour et al., 2021). In this review, we provide a systematic and critical examination of MOF-based pesticide delivery technologies, with particular emphasis on their structural advantages, synthesis strategies, functional modifications, and environmentally responsive release behaviors. MOF-enabled pesticide nanoformulations are categorized into four major classes, namely insecticides, fungicides, herbicides, and plant growth regulators. Representative systems are summarized, and key structure-performance relationships as well as their relevance to field applications are elucidated. Finally, the major challenges that limit further development are identified, and future research opportunities are outlined to support the rational design of next-generation MOF-enabled agricultural technologies for sustainable crop protection (**Figure 1**).

## 2 Preparation and functional modification of metal-organic framework

**Figure 2:**
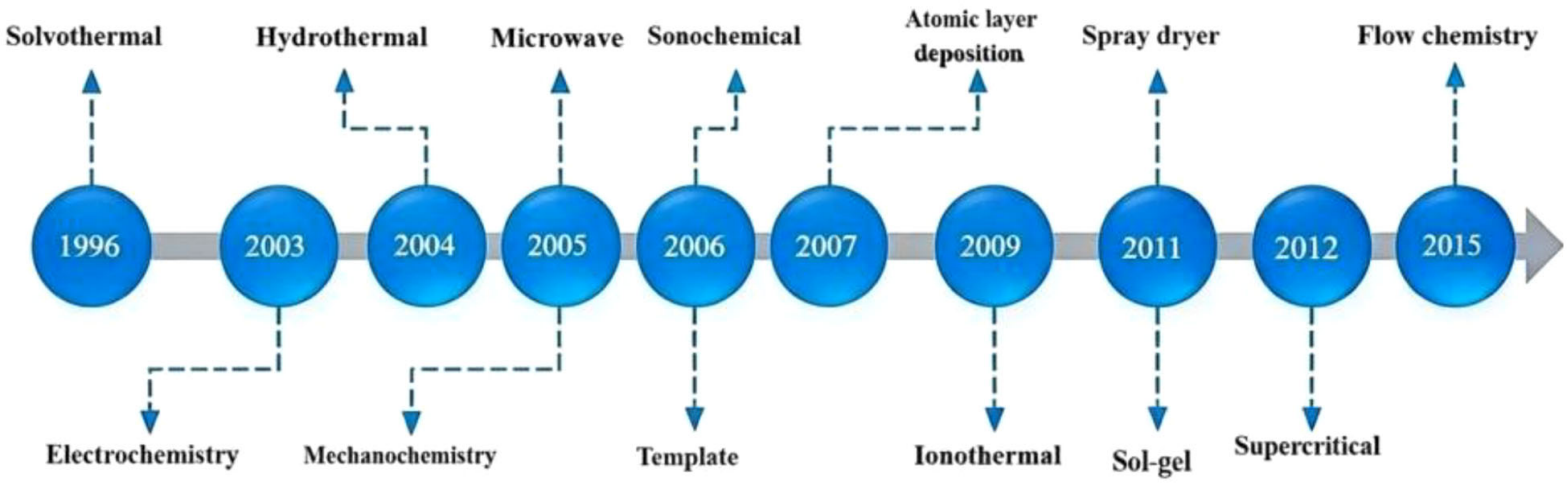
Timeline of the development of MOF synthesis methods.

The MOFs constitute a rapidly expanding class of crystalline porous materials distinguished by their tunable pore structures, exceptionally high surface areas, abundant coordination sites, and excellent thermal and chemical robustness (Guo et al., 2025). By tailoring metal node connectivity, ligand functionality, and topological geometry, researchers have developed a series of representative MOF families-including zeolitic imidazolate frameworks (ZIFs), Materials of Institute Lavoisier (MILs), isoreticular metal-organic frameworks (IRMOFs), porous coordination networks (PCNs), University of Oslo (UiO) frameworks, and porous coordination polymers (PCPs) (Sampaio et al., 2025). The tunable architecture of MOFs grants them exceptional versatility, supporting their extensive use in catalysis, environmental engineering, biomedicine, and agricultural applications (Díaz-Ramírez et al., 2025; Ghaedi et al., 2025; Li et al., 2024). **Figure 2** shows the timeline of the most common synthetic methods developed over the past two decades. The properties and functions of MOFs are intrinsically governed by their synthetic routes, with solvothermal/hydrothermal synthesis, dry-gel conversion, and microwave-assisted methods constituting the most commonly employed strategies (Nguyen et al., 2024; Ravipati et al., 2023; Yan et al., 2024). By precisely tuning critical parameters such as the metal-to-ligand ratio, solvent polarity, pH values, and reaction temperature and time, MOFs with well-defined particle sizes, pore architectures, crystallinity, and surface chemistries can be reproducibly obtained (Cucinotta et al., 2025; Shaari et al., 2025). These controllable physicochemical features not only dictate the intrinsic behavior of the materials but also provide a versatile basis for tailoring MOFs to the requirements of specific application scenarios.

Although MOFs have proven highly effective in areas such as gas storage, catalysis, drug delivery, and chromatographic separation, their application in pesticide delivery remains at an early stage of development (Li et al., 2022; Mirzaei et al., 2024; Ojaghzadeh Khalil Abad et al., 2023; Serag et al., 2025). Nevertheless, several representative MOF families, including ZIFs, MILs, UiOs and PCNs, have emerged as particularly promising candidates for agricultural applications (Chen et al., 2024; Fang et al., 2025; Hou et al., 2025; Li et al., 2025). Their highly ordered porous networks, large pore volumes, and tunable coordination environments enable superior pesticide encapsulation efficiency, enhanced molecular compatibility, and improved dispersibility relative to traditional inorganic or polymeric carriers (Liu et al., 2023; Masood et al., 2025). In addition, MOFs constructed through the coordination of metal ions and organic ligands exhibit highly designable structural features, with the stability of coordination bonds, accessibility of pore channels, and the chemical state of surface functional groups all responding sensitively to external environmental stimuli (He et al., 2021). Previous studies have demonstrated that the framework structures of MOFs can undergo reversible or partially reversible adjustments under changes in pH, temperature, light irradiation, redox, or magnetic fields (Hou et al., 2024; Hu et al., 2024; Meng et al., 2021; Wan et al., 2022; Zong et al., 2023). Such adjustments may involve ligand dissociation, pore expansion, framework flexibility transitions, or surface chemical reorganization. These structural variations directly influence the diffusion behavior, binding affinity, and spatial confinement of guest molecules within the pores, thereby providing controllable mechanisms for the directional release of pesticide molecules (Feng et al., 2022). To enhance their performance in agrochemical delivery, researchers have developed diverse synthesis routes and functional modification strategies aimed at improving loading capacity, structural stability, and environment-responsive release behavior.

### 2.1 Synthesis methods of MOFs

The synthesis of MOFs relies on the controlled coordination between metal nodes and organic ligands, enabling precise regulation of particle size, pore structure, morphology, and functional properties. These structurally tunable characteristics make MOFs particularly suitable for controlled pesticide delivery (Kazemi et al., 2025; Singh et al., 2024). Among the various synthesis strategies, hydrothermal/solvothermal methods and one-pot assembly are the most widely employed and hold the greatest potential for future industrial-scale production (Yang et al., 2024; Zhang et al., 2025).

#### 2.1.1 Hydrothermal/solvothermal method

**Figure 3:**
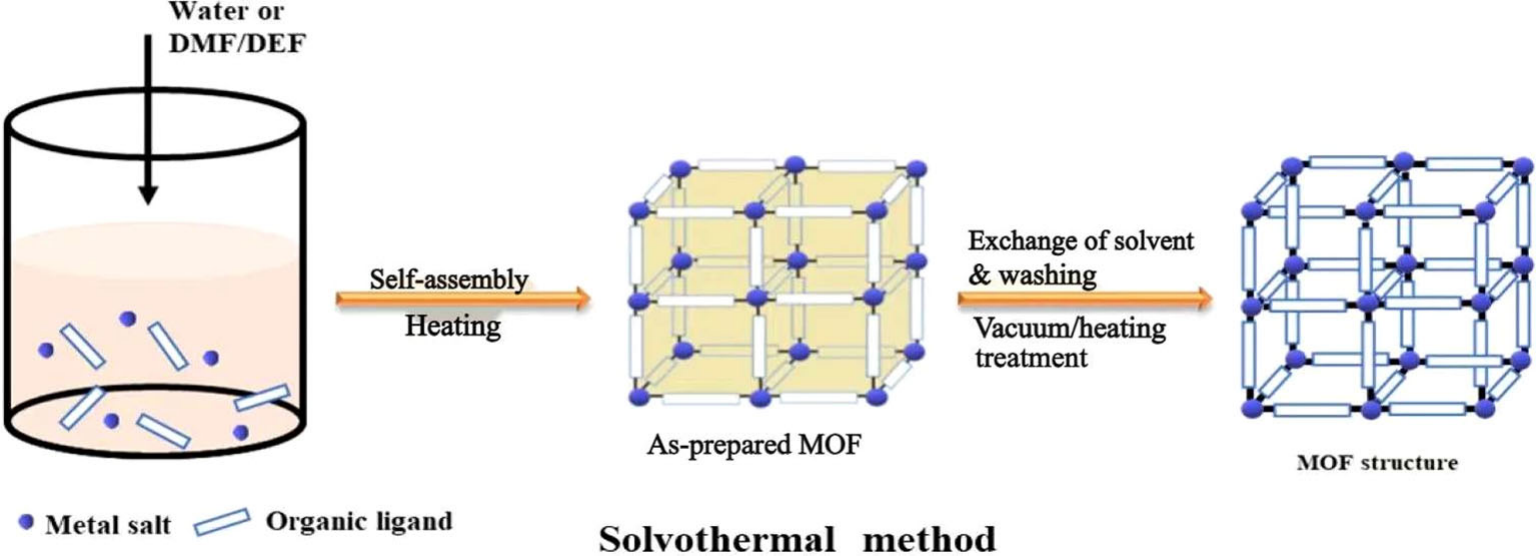
The schematic diagram of MOF structure synthesized by hydrothermal/solvothermal method. Reprinted with permission from ref (Yusuf et al., 2022). Copyright 2022, American Chemical Society.

The hydrothermal/solvothermal method is one of the most established and versatile strategies for synthesizing MOFs (Swain et al., 2025). In this approach, metal salts and organic ligands are dissolved in a polar solvent and sealed within a high-pressure autoclave, where elevated temperature and autogenous pressure promote coordination and framework assembly (Chongdar et al., 2022). The synergistic effects of high temperature, solvent polarity, and pressure enhance the solubility and reactivity of precursor species, including those that are poorly soluble under ambient conditions, thereby facilitating efficient metal-ligand coordination (**Figure 3**). As a result, this method reliably produces MOFs with high crystallinity, well-defined morphology, and strong structural stability (Mishra et al., 2024). The crystallization process is highly tunable, and key operational parameters such as reaction temperature and duration, pH, solvent composition, precursor stoichiometry, and the presence of surfactants or polymers determine nucleation kinetics, particle size, pore characteristics, and crystal morphology (Yusuf et al., 2022). Such tunability makes this method particularly valuable for designing MOFs with tailored porosity or particle dimensions suitable for pesticide encapsulation.

Shan et al. synthesized Fe-MIL-100 via a hydrothermal reaction using Fe^3+^ and 1,3,5-benzenetricarboxylic acid at 150°C, obtaining well-defined octahedral crystals with a high specific surface area (2251 m^2^ g^-1^) and stable porous architecture (Shan et al., 2020). Similarly, Wan et al. prepared UiO-66 through a solvothermal reaction of ZrCl_4_ and terephthalic acid at 120°C for 24 h, yielding uniform spherical nanocrystals characterized by a high surface area (1021.79 m^2^ g^-1^) and large pore volume, indicative of a well-developed microporous/mesoporous network (Wan et al., 2022). Zhu et al. synthesized PCN-222 via a solvothermal route using ZrOCl_2_·8H_2_O and TCPP at 120°C, producing needle-like nanocrystals with high crystallinity and large specific surface area. The framework exhibited excellent structural stability in aqueous media but underwent controlled degradation under alkaline conditions, reflecting its tunable coordination environment and pH-responsive structural adaptability (Zhu, H. et al., 2025). Fang et al. prepared a CuS@Cu-MOF composite via a hydrothermal process followed by *in situ* sulfurization, yielding spindle-shaped porous frameworks decorated with CuS nanoparticles. The introduction of CuS preserved the intrinsic porosity of the parent Cu-MOF and produced a hierarchical structure with broader pore channels and enhanced stability, thereby improving its efficiency as a pesticide-loading carrier (Fang et al., 2024).

#### 2.1.2 One-pot method

**Figure 4:**
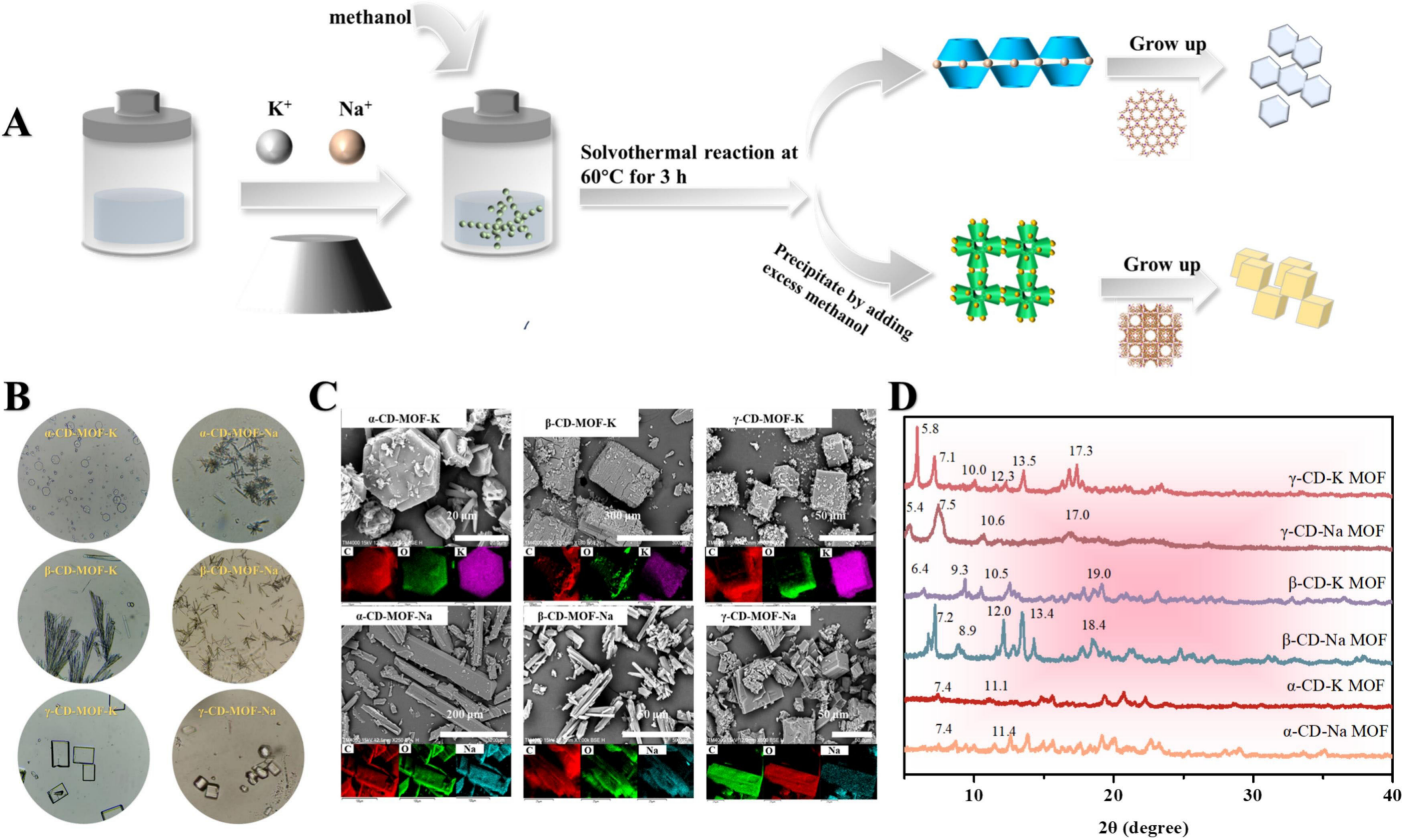
Schematic diagram of synthesis of CD-MOFs by one-pot hydrothermal method **(A)**, optical morphology **(B)**, SEM images **(C)**, and XRD patterns **(D)** of CD-MOFs. Reprinted with permission from ref (Chen et al., 2025a). Copyright 2025, Elsevier.

The one-pot method provides a simple, scalable, and energy-efficient strategy for synthesizing MOFs, particularly those belonging to the ZIF family (Sarfudeen et al., 2025). In this approach, metal ions and organic ligands are mixed directly in a single reaction vessel, where coordination reactions occur spontaneously without the need for intermediate purification (**Figure 4**). This streamlined synthetic process significantly reduces reaction time, solvent consumption, and operational complexity, making the method attractive for large-scale preparation (Perveen et al., 2025). The one-pot method is especially effective for ZIF-type MOFs because of the rapid coordination kinetics between metal ions and imidazolate ligands (Tsai et al., 2019). Fine-tuning parameters such as solvent polarity, reactant concentration, ligand-to-metal ratio, and stirring conditions allows control over particle size, dispersibility, and morphology.

Liang et al. adopted a one-pot assembly of Zn^2+^, 2-methylimidazole, prochloraz, and dopamine to construct a light-triggered, pH-responsive ZIF-8 nanocarrier with uniform morphology and strong framework stability (Liang et al., 2021). Essential-oil-based delivery systems have also been fabricated through one-step coordination between plant essential oils, Zn^2+^, and 2-methylimidazole, forming EO@ZIF-8 nanoparticles with enhanced encapsulation and volatilization resistance (Ding et al., 2023). Likewise, dazomet-loaded ZIF-8 was obtained through a similar one-step self-assembly process, enabling soil-condition-triggered release (Ren et al., 2022). An et al. further expanded the applicability of the one-pot strategy by developing a series of pH-responsive NKMOF-101 materials from squaric acid and various divalent metal ions (Mg^2+^, Mn^2+^, Zn^2+^, Co^2+^, Ni^2+^). The resulting cubic NKMOF-101 crystals possessed high crystallinity, tunable composition, and an accessible pore network conducive to azoxystrobin incorporation, achieving high loading capacities (up to 0.14 mg mg^-^¹) and encapsulation efficiencies of approximately 90%. The NKMOF-101 framework also allowed pH-responsive structural degradation, providing a controllable environment for pesticide release and demonstrating strong potential for further functionalization (An et al., 2025). Dong et al. also demonstrated the effectiveness of the one-pot approach by embedding pyrethrin directly into ZIF-8 during nucleation, producing uniformly sized Py@ZIF-8 nanoparticles with stable microporosity (Dong et al., 2025). Across these systems, the one-pot route consistently facilitated intimate host-guest integration and yielded MOFs with tunable pore structures, improved physicochemical stability, and strong affinity toward pesticide molecules.

#### 2.1.3 Vapor diffusion method

**Figure 5:**
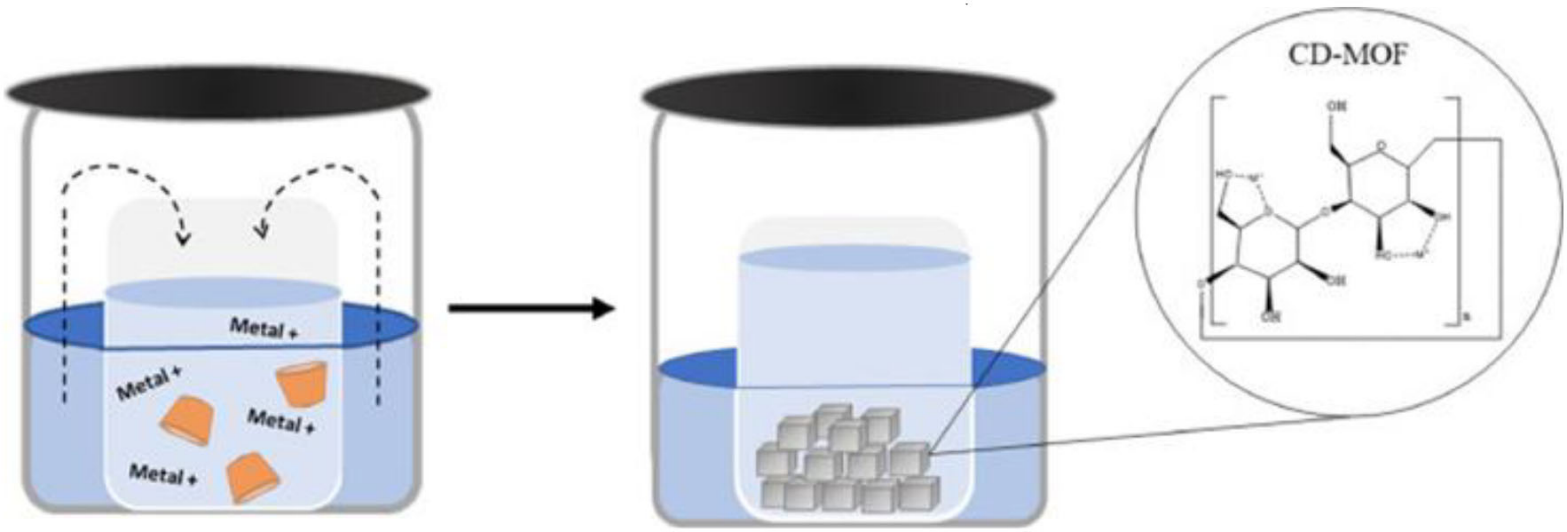
Schematic illustration of vapor diffusion technique to obtain γ-CD-MOF crystals. Reprinted with permission from ref (Abuçafy et al., 2024). Copyright 2024, Elsevier.

The vapor diffusion method is a widely used technique for synthesizing cyclodextrin-based MOFs (CD-MOFs) (Abuçafy et al., 2024; Sadeh et al., 2023). In this approach, cyclodextrins and potassium salts are dissolved in water to form a homogeneous precursor solution, which is then exposed to a volatile organic antisolvent, typically ethanol, methanol, or isopropanol (Xu et al., 2023). As the organic vapor gradually diffuses into the aqueous phase, the solubility of cyclodextrin–metal complexes decreases in a controlled manner, promoting slow supersaturation, nucleation, and orderly crystal growth (**Figure 5**). Owing to its mild conditions, simplicity, and high solvent efficiency, vapor diffusion has become the predominant route for preparing γ-CD-MOF and β-CD-MOF materials.

Key parameters influencing γ-CD-MOF formation include the type and concentration of potassium salts, the polarity and volatility of the diffusing solvent, the vapor diffusion rate, and the initial concentration of γ-cyclodextrin. These factors collectively regulate the crystallization kinetics, framework symmetry, particle size, and pore accessibility of the resulting MOFs (Pan et al., 2022). Chen et al. prepared γ-CD-MOF using γ-cyclodextrin and KOH under ethanol vapor diffusion, obtaining uniform cubic crystals with well-defined porosity, suitable for encapsulating avermectin (Chen et al., 2025b). Zheng et al. further demonstrated that altering the potassium source (KCl, KAc, K_2_CO_3_, etc.) significantly modulates crystal morphology and pore environments, thereby improving host-guest interactions and enhancing pesticide loading performance (Zheng et al., 2023). Similar vapor-diffusion-assembled γ-CD-MOFs have been used to encapsulate thymol, curcumin, and other hydrophobic compounds, benefiting from the large hydrophobic cavities and high structural integrity inherent to CD-based frameworks (Min et al., 2024; Nong et al., 2025; Yu et al., 2025). Liu et al. also reported β-CD-MOF crystals formed through analogous vapor-phase assembly, providing high adsorption capacity and stable pore networks suitable for pesticide delivery (Liu et al., 2019).

### 2.2 Functional modification of MOFs

MOF-based nano-pesticides have emerged as promising candidates for sustainable agricultural applications due to their ability to reduce pesticide consumption, enhance control efficacy, and improve environmental safety. However, unmodified MOFs typically exhibit passive and non-targeted release behavior, often leading to rapid initial release and insufficient late-phase availability. Such limitations hinder their effectiveness in meeting the spatiotemporal requirements of pest control. To address these challenges, functional modification of MOFs has become essential for enabling environmentally responsive, precise, and on-demand pesticide delivery. Through structural modulation, post-synthetic etching, or composite engineering, MOFs can acquire additional binding sites, improved environmental stability, and tailored stimulus-responsive release characteristics. The major functionalization strategies are summarized as follows.

#### 2.2.1 Coordination modulation

Coordination modulation takes advantage of the intrinsic structural tunability of MOFs by introducing secondary metal ions or competing ligands during synthesis to regulate coordination environments and pore architectures. Through controlled competition at metal–ligand binding sites, this strategy adjusts nucleation kinetics and the equilibrium between bond formation and dissociation, thereby generating frameworks with additional active sites, tailored defects, and refined pore structures. Such modifications increase the binding affinity for pesticide molecules and enable more precise control over loading and release behavior.

Yang et al. demonstrated this approach by incorporating Co^2+^ into a Zn and 2-methylimidazole system, where metal–metal competition reconfigured the coordination network, producing bimetallic Zn/Co-ZIFs with enhanced porosity, structural robustness, and improved pesticide adsorption capacity (Yang et al., 2025). A similar effect was observed in MPN-modulated ZIFs, where polyphenolic ligands competed for coordination with metal centers, introducing controlled defects and increased hydrophilicity, ultimately leading to higher pesticide encapsulation and environmentally responsive release (Sheng et al., 2025). Coordination modulation has also been applied to bimetallic MOF hybrid platforms, in which the incorporation of ions such as Fe^3+^and Cu^2+^ shifted coordination equilibria during crystal growth, yielding frameworks with dual-stimuli responsiveness, higher densities of active sites, and stronger host-guest interactions conducive to sustained pesticide delivery (Hegde et al., 2025).

#### 2.2.2 Post-synthetic modification

Post-synthetic modification (PSM) offers a flexible and powerful route for engineering MOFs after the primary framework has been assembled, enabling precise control over surface chemistry, interfacial characteristics, and environmental responsiveness while maintaining the structural integrity of the parent lattice. Unlike coordination modulation, which regulates bond formation during nucleation, PSM introduces functional components post-assembly through polymer grafting, biopolymer capping, MOF-on-MOF overgrowth, or supramolecular gating. These modifications enhance water stability, reduce premature pesticide leakage, introduce multi-stimuli responsiveness, and improve the applicability of MOF-based systems under realistic agricultural conditions.

One important category of PSM involves polymer and biopolymer functionalization, which provides degradable gatekeeping layers to regulate molecular diffusion and impart biochemical or pH-triggered release behavior. For instance, NH_2_-MIL-101(Fe) modified with D-cellobiose and carboxymethyl cellulose (MIL@A@D@C) forms a polysaccharide-based shell that responds synergistically to cellulase and acidic microenvironments, enabling precise fungicide release at maize infection sites (Li et al., 2025). Likewise, tannic-acid coated Fe-MOFs utilize Fe^3+^-tannic acid coordination networks as responsive caps capable of activating antifungal agents only under pathogen-relevant biochemical stimuli (Dong et al., 2021). Long-acting insecticidal systems such as DNF@ZIF-8@PMMA/zein further demonstrate how polymerization followed by biopolymer deposition can establish robust multilayer shells that suppress premature leakage while supporting protease-triggered activation within insect digestive tracts (Ma et al., 2022). A second PSM route involves inorganic or organic shell deposition, in which external layers improve aqueous stability and adjust pesticide release kinetics. The CAP@MIL-101(Fe)@SiO_2_ system exemplifies this approach, as the silica shell enhances moisture resistance, moderates the initial burst effect, and facilitates pH-dependent release (Gao et al., 2021a). PSM also enables the construction of MOF-on-MOF heterostructures, allowing complementary integration of framework properties. In the Z8@Z90 system, a hydrophilic ZIF-90 shell is post-synthetically grown on ZIF-8, resulting in improved dispersion stability and accelerated degradation under pathogen-induced acidic conditions, which in turn enhances tebuconazole loading and pathogen-specific release (Chang et al., 2024).

## 3 Application of MOFs in pesticide delivery

In recent years, MOF-based pesticide delivery systems have gained growing attention for their versatility in agricultural applications. Based on the nature of the active ingredient and its biological target, current MOF nanoformulations can be classified into MOF-based insecticides (**Table 1**), fungicides (**Table 2**), herbicides (**Table 3**), and plant growth regulators.3.1 MOF-based insecticides.

**Table 1:** MOF-based insecticides for insect control.

MOF	Average particle size (nm)	BET(m^2^g^–1^)	Compositions	Loaded insecticides	Activity studies	Pests	Ref.
MIL-101(Fe)	270	2383.10	Silica	Chlorantraniliprole (CAP)	The mortality of the CAP@MIL-101(Fe)@silica treatment (86%) against *P. xylostella* larvae was significantly higher than that of the CAP suspension concentration treatment (36.7%).	*Plutella xylostella larvae*	(Gao et al., 2021)
ZIF-8	200	1775	Poly(methyl methacrylate) (PMMA), zein	Dinotefuran (DNF)	The insecticidal efficacy of DNF reduced to 25.6% on the 14th day of application, the insecticidal efficacy of the MOF hybrid remained above 66%.	Mole cricket (*Gryllotalpaspps*)	(Ma et al., 2022)
ZIF-8	150	1153.15		β-cypermethrin (β-CYP)	After 14 days, the mortality rate for the high concentration of β-CYP/ZIF-8 treatment (8 mg a.i./L) was still over 70% higher than commercial WP and EC, which decreased to 41.7 and 45.0%.	Termites (*Coptotermes formosanus* Shiraki)	(Ma et al., 2022)
UiO-66-(COOH)_2_	150	344.68	Poly(N-isopropyl acrylamide) (PNIPAm)	Indoxacarb (IDC)	After 48h, the IDC@UiO-66-(COOH)_2_-PNIPAm group shows a better antipest activity(83%) than the IDC group(77%).	*Spodoptera frugiperda*	(Wan et al., 2022)
MIL-101(Fe)-NH_2_	161.54	697.31	Carboxymethyl starch (CMS)	Chlorantraniliprole (CAR)	The *LC_50_* values of CAR suspension concentrate and CAR@MIL-101(Fe)-CMS nanoparticles against *Spodoptera frugiperda* larvae were 0.91 mg/L and 0.99 mg/L respectively.	*Spodoptera frugiperda*	(Liang et al., 2022b)
γ-CD–MOFs	2562	380.90		Avermectins (AVM)	The *LC_50_* values of AVM−γ-CD–MOF wettable powders (MWP) and VM TC wettable powders against adult females were 0.26 mg/L and 5.342 mg/L respectively.	*Panonychus citri (McGregor)*	(Chen et al., 2023a)
ZIF-90	200-300	857.19	Mono-(6-amino-6-deoxy)-β-cyclodextrin (β-CD-NH_2_)	*Indoxacarb (IDC)*	The mortality rate was higher than that of the IDC at 48 h and 72 h of treatment.	Red imported fire ant (RIFA)	(Yang et al., 2023)
MIL-101(Fe)-NH_2_	129.82		Sodium lignosulfonate (SL)	Chlorfenapyr (CF)	During 24 to 72 h of treatment, the *LC_50_* values of the CF-SC and CF@MIL-101-SL treatments were 1.59 to 4.26 mg/L and 0.53 to 2.22 mg/L.	*Spodoptera frugiperda*	(Chen et al., 2023b)
PCN-777	250	725	Sodium lignosulfonate (SL) and chitosan (CS)	Avermectin (AVM)	The median lethal concentration (*LC_50_*) of AVM@PCN-777@SL/CS against S. litura was 25.517 mg L^–1^, which was lower than the respective *LC_50_* values of AVM SC (31.039 mg L^–1^) and AVM TC (29.645 mg L^–1^).	*Spodoptera litura* Fabricius	(Liu et al., 2023)
β-cyclodextrin-doped ZIF-8 (β-CD/ZIF-8)	60	190.21	Silica	Imidacloprid (IMI)	The median lethal concentration (*LC_50_*) values of IMI@β-CD/ZIF-8@ST against *Plutella xylostella* is calculated to be 30.32 mg/L, which is lower than the respective *LC_50_* values of IMI (44.54 mg/L) and IMI WG (34.85 mg/L).	*Plutella xylostella*	(Wan et al., 2024)
ZIF-8	1000	954.14		*Cry1Ac*	Cry1Ac@ZIF-8 prolonged the effective insecticidal period on maize leaves from <7 days (Cry1Ac) to ≥7 days, maintaining 25.44% corrected mortality, whereas Cry1Ac rapidly lost activity despite a slightly higher initial mortality.	Asian corn borer	(Lu et al., 2024)
ZIF-8	120		Chitosan (CS)	Double-stranded RNA (dsRNA) and cycloxaprid (Cyp)	The mortality rate of the IDC@ZIF-90-CD-treated group was higher than that of the IDC EC-treated group at same time.	*Diaphorina citri*	(Wang et al., 2025)
ZIF-8	280–400	1327.314		Emamectin benzoate (EB)	The *LC_50_* of EB@ZIF-8 was 52.503 mg/L, showing a 1.921-fold increase compared to the *LC_50_* of EB against nematodes (100.893 mg/L) after 24 h.	*Bursaphelenchus xylophilus*	(Teng et al., 2025)
UiO-67	453.37 ± 1.97			Dinotefuran (DNF)	The *LC_50_* of 98% DNF technical against pea aphids was 63.074 mg/L, the *LC_50_* of DNF@UiO-67 was 58.578 mg/L.	*Pea aphids*	(He et al., 2025)

**Table 2:** MOF-based microbicides for phytopathogen control.

MOF	Average particle size (nm)	SBET(m^2^g^–1^)	Compositions	Loaded fungicides	Activity studies	phytopathogen	Ref.
ZIF-67	350			Boscalid (Bos)	The control efficacies of Boscalid (WG) and Boscalid@ZIF-67 at the same dosage were calculated to be 83.9% and 100%, respectively.	*Botrytis cinerea*	(Zhang et al., 2022)
ZIF-8	71.5	1228.87		Dazomet (DZ)	DZ@ZIF-8 displayed favorable persistency against the pathogen (74.8 ± 1.8%) 10 d after initial inoculation, which was 42.7% higher than that of DZ (52.4 ± 0.8%).	*Botrytis cinerea*	(Ren et al., 2022a)
UiO-66	238.62	221.64	Hydroxypropyl cellulose (HPC)	Pyraclostrobin (PYR)	The EC50 value (**Table 2**) of PYR@UiO-66@HPC was determined to be 0.276 mg/L, which is higher than that of PYR-TC (0.251 mg/L) and lower than that of BASF-CS (0.289 mg/L).	*Rhizoctonia solani*	(Ma et al., 2023)
ZIF-8	841.7	1527		Prochloraz (Pro) and Small interfering RNA (siRNA)	The IC_50_ value of Pro/DA-6 @ZIF-8 @siRNA was 0.09 ± 0.004 mg/L, which is dramatically reduced by 52.6% relative to Pro technical (0.19 ± 0.05 mg/L).	*Rhizoctonia solani*	(Huang et al., 2023)
benzimidazole-modified NH_2_-MIL-101(Fe)	514.8	559.0	β-cyclodextrin	Osthole	β-CD@B-MIL-101(Fe)-OS completely suppressed soft rot development on tomatoes after 96 h, whereas severe rot symptoms were observed in the control group.	*Botrytis cinerea*	(Wang et al., 2023)
Cu@ZIF-8	80.38			Fludioxonil (Flu)	The half-maximal effective concentration (*EC_50_*) of Flu TC and Flu@Cu@ZIF-8 was 0.041 mg L^−1^ and 0.038 mg L^−1^.	*Fusarium graminearum*	(Xu et al., 2023)
UiO-66-NH_2_	527.1	393.22	Halloysite nanotubes (HNTs)	Pyraclostrobin (Pyr)	Upon the effective concentration of Pyr at 10 mg/L, the inhibition rates of Pyr, Pyr@HNTs and Pyr@NH_2_-Uio-66 MOFs@HNTs against the strawberry grey mould reached 55.8%, 62.8% and 67.4%.	Strawberry gray mold	(Zhu et al., 2024)
ZIF-8	397 ± 4.50	1457.56	Fe^3+^ and gallic acid (GA) metal-phenolic network	Hexaconazole (Hex)	The *EC_50_* of Hex EC, ZIF-8@Hex and ZIF-8@Hex@Fe/GA were 0.1724 mg/L, 0.1308 mg/L and 0.0938 mg/L.	*Phomopsis* sp.	(Hu et al., 2024)
ZIF-8	131.1	1814.5315	ZIF-90	Tebuconazole (TEB)	TEB SC, T@Z8, and T@Z8@Z90 and their respective concentrations, with *EC_50_* values recorded as 0.0838, 0.1097, and 0.0554 mg/L.	*Sclerotium rolfsii*	(Chang et al., 2024)
Cu-TCPP	13.6	415.9	Fe^3+^ and tannic acid (TA) metal-phenolic network	Diniconazole (DIN)	At a DIN concentration of 10 mg mL^-1^, the inhibition rates of DIN TC and Cu-TCPP@DIN@MPN against Fusarium wilt were 63.6% and 90.9%, respectively.	*Fusarium oxysporum*	(Han et al., 2024)
UiO-66	163	818.75	Zn^2+^ and tannic acid (TA) metal-phenolic network	Ipconazole (IPC)	The nano-pesticide-loaded IPC@UiO-66-TA-Zn^2+^ nano flowable suspension exhibited improved control of rice bakanae (*Fusarium fujikuroi*), with efficacy rates ranging from 84.09% to 93.10%, compared with IPC flowable suspension, which showed control efficacies of 81.82% to 84.48%.	*Fusarium fujikuroi*	(Zhang et al., 2024)
UIO-66-NH_2_	60	1223.6	Carboxymethyl cellulose (CMC), Chitosan quaternary ammonium salt (CQAS)	Boscalid (Bos)	The median effective concentrations (*EC_50_*) for Bos, Bos WG, and Bos nanopesticide were 0.0903, 0.8361, and 0.1442 mg L^-1^ after 72h.	*Sclerotinia sclerotiorum*	(Yang et al., 2025)
Amino-doped ZIF-8	144.25	1059.2034	Pectin	Pyraclostrobin (Pyr)	The *EC_50_* values of PYR nanopesticide and Pyr SC were 0.0582 mg L^-1^ and 0.0900 mg L^-1^, respectively.	*Fusarium graminearum*	(Zhu S, et al., 2025)
ZIF-8	83	1932.63	Polydopamine (PDA)	Boscalid (Bos)	The EC50 values of the Bos WP group and the Bos@ZIF-8@PDA group on day 7 were 0.101 mg/L and 0.092 mg/L.	*Botrytis cinerea*	(Zhang et al., 2025)
Ti-MOF@Diatomaceous earth (DE),		731	pectin (Pec)	Prochloraz (Pro)	Pec@Pro@Ti-MOF@DE exhibited a control efficacy of 95.6 ± 0.6% against gray mold on fruits, significantly outperforming prochloraz (82.8 ± 1.8%).	*Botrytis cinerea*	(Bhat et al., 2025)

**Table 3:** MOF-based herbicides for weeds control.

MOF	Average particle size (nm)	SBET(m^2^g^–1^)	Loaded Herbicides	Compositions	Activity studies	Weeds	Ref.
ZIF-8	60 and 113		DiS–NH_2_, DiS-*O*-acetyl	2-hydroxypropyl-β-cyclodextrin (HP-β-CD)	The herbicidal performance of MOF@DiS–NH_2_ and MOF@DiS-O-acetyl surpassed that of Logran, which was employed as a positive control in the bioassay.	*Lollium rigidum* Gaudin*, Echinochloa crus-galli (*L.*) and Amaranthus Viridis*	(Mejías et al., 2021)
GR-MOF-7	223		Glufosinate		Active concentration of glufosinate (0.01 M), GR-MOF-7 fully inhibited the seed germination (100 ± 0% of seed germination reduction), showing a greater herbicide effect than the free glufosinate (32 ± 7%)of seed germination reduction.	*Raphanus sativus*	(Sierra-Serrano et al., 2022)
ZIF-67	470	1038	Pretilachlor (Pre), 4-(Dichloroacetyl)-1-oxa-4-azospiro[4,5]decane (AD-67)		At an application rate of 100 g a.i. ha^-1^, AD-67@Pre@ZIF-67 achieved a visual control efficacy of 89%, which was approximately twofold higher than those of Pre and Pre: AD-67 (3:1).	*Echinochloa crus-galli* (L.) P. Beauv.	(Guo et al., 2023)
MIL-101(Fe)	252	1822	Paraquat (PQ)	Carboxymethyl cellulose-Ca^2+^ (CMC-Ca^2+^)	Technical PQ showed time-dependent efficacy at low dosage, whereas PQ@MIL-101(Fe³^+^)@CMC-Ca²^+^ achieved rapid and dose-enhanced weed control through sustained release of PQ.	*Cynodon dactylon*	(Dong et al., 2023)
ZIF-8 and Fe_3_O_4_			Diuron (DU) and p-sulfocalix[4]arenedicarboxylicacid (SCX4)		Direct application of the DU solution caused rapid plant death within 7 days, whereas MOF-based composites markedly reduced plant mortality.	*Chenopodium album* L.	(Saklan et al., 2024)
GR-MOF-20			Glyphosine (H5Gly)		After 7 days, H5Gly (200 mg L^–1^) was able to inhibit 13.3 ± 2.9% of invasive grass seed germination, the MOF (450 mg L^–1^) was able to inhibit 21.7 ± 2.9%.	*Lolium multiflorum*	(Contreras et al., 2025)
MIL-101(Cr)	303	1225	Paraquat (PQ)	MoS_2_, Fe_3_O_4_, chitosan (CS)	The herbicidal efficacy of PQ 0.25( mg mL^−1^ )increased to 86.9%,the herbicidal efficacy of PQ@MMF@CS(1.5 mg mL ^−1^)increased to 97.4%.	*Cynodon dactylon*	(Cao et al., 2025)
ZIF-8	118.46	1451.08	Bispyribac-sodium (BIS)		The BIS@ZIF-8 treatment showed significantly higher herbicidal activity than BIS under natural conditions.	*Echinochloa crus-galli* (L.) P. Beauv.	(Zhang et al., 2025)
ZIF-8	162.4	1526.8	2,4-D		At a given concentration, 2,4-D@ZIF-8 exhibited higher herbicidal activity than technical 2,4-D. In addition, ZIF-8 alone inhibited the growth of barnyard grass in a concentration-dependent manner.	*Echinochloa crus-galli* (L.) P. Beauv.	(Cai et al., 2025)
MIL-101(Fe)	280	1822	Paraquat (PQ)	Carboxymethyl cellulose-Ca^2+^ (CMC-Ca^2+^)	PQ@MIL-101(FeIII)@CMC-CaII nanoherbicides, the control efficacy was ca. 31.2% and 66.2% at the dose of 1.0 and 1.5 mg mL−1 respectively after 1 day of spraying; the control efficacy achieved ca. 75.6% and 92.8% at the dose of 1.0 mg mL−1, and achieved ca. 97.8% and 99.0% at the dose of 1.5 mg mL−1after 4 and 8 days of spraying.	*Cynodon dactylon*	(Dong et al., 2023)
UiO-66-NH_2_	194.92	962.36	Paraquat (PQ)	β-cyclodextrin, cinnamic acid derivatives	Sunlight-activated PQ@MOF@CC@CD matches free paraquat’s herbicidal efficacy.	*Echinochloa crus-galli* (L.) P. Beauv.	(Li et al., 2025)

**Figure 6:**
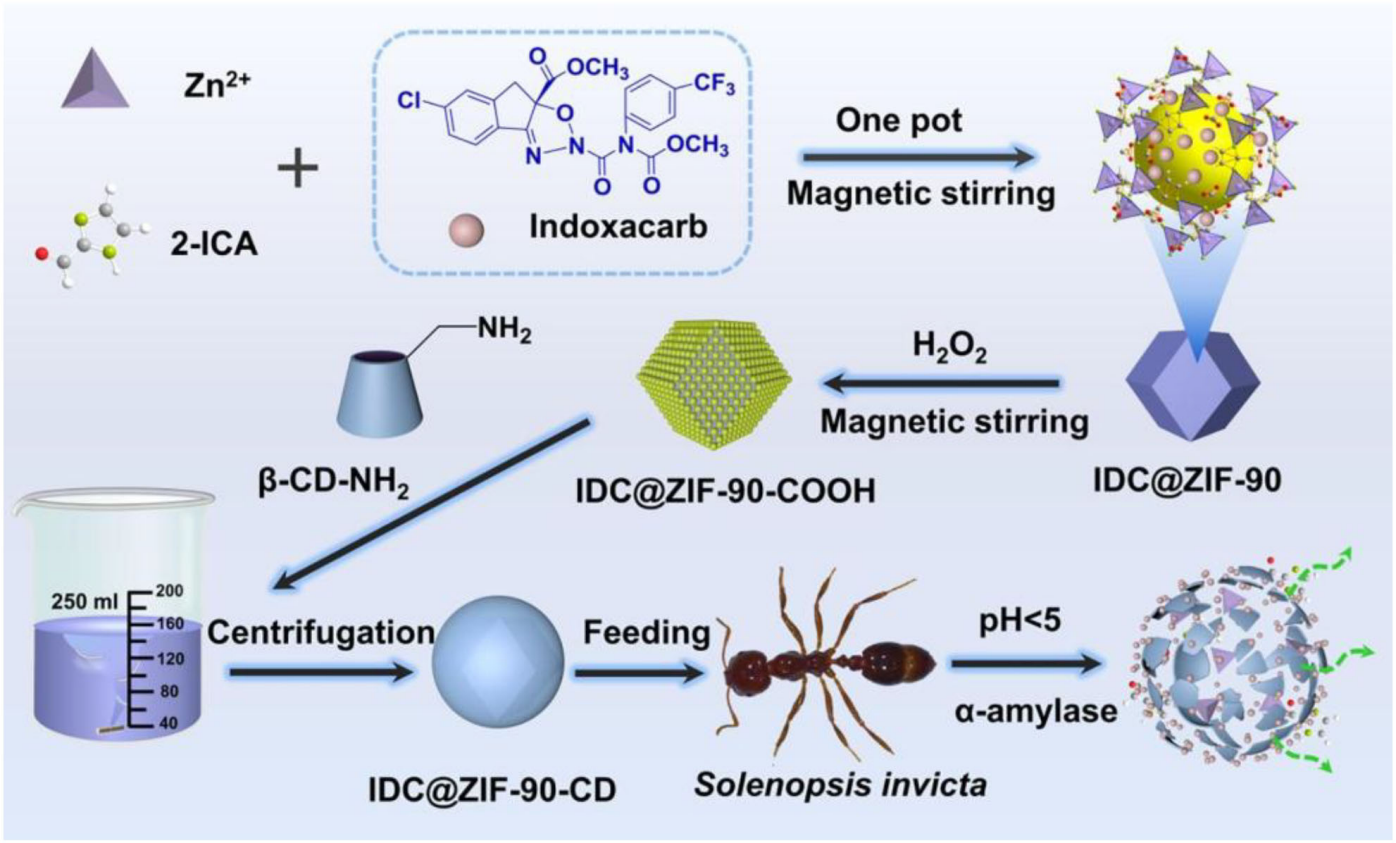
The steps used to synthesize IDC@ZIF-90-CD and its application in the intelligent control of red imported fire ants. Reprinted with permission from ref (Yang et al., 2023). Copyright 2023, Elsevier.

MOFs have emerged as a highly versatile class of nanocarriers for insecticidal delivery owing to their tunable porosity, structural stability and capacity for microenvironment-responsive release. Across multiple MOF systems, recent research has demonstrated substantial enhancements in loading capacity, environmental tolerance and insecticidal efficacy, together with reduced toxicity to non-target organisms. For example, indoxacarb-loaded ZIF-90 modified with mono-(6-amino-6-deoxy)-β-cyclodextrin yielded IDC@ZIF-90-CD nanoparticles of approximately 249 nm with an 18.43% loading efficiency. The β-cyclodextrin gate significantly inhibited premature leakage and photodegradation, while acidic pH and α-amylase, simulating the digestive environment of *Solenopsis Invicta*, triggered rapid release (**Figure 6**). Correspondingly, the nanoformulation induced severe peritrophic membrane disruption, extensive epithelial shedding and marked perturbation of amino acid metabolism, achieving mortality rates more than three times higher than the technical indoxacarb product (Yang et al., 2023).

Similar performance enhancements have been observed for MOF-stabilized biopesticides. Cry1Ac@ZIF-8 prepared via biomimetic mineralization preserved the tertiary structure of Cry1Ac and improved thermal and UV resistance, retaining over 90% activity after heat stress and exhibiting markedly prolonged foliar persistence. The nanocarrier enhanced Cry1Ac penetration and retention on maize leaves and maintained strong larvicidal activity toward *Ostrinia furnacalis*, *Helicoverpa armigera* and *Spodoptera litura* relative to the unencapsulated protein (Lu et al., 2024). Hybrid MOF–polymer systems have further expanded the scope of insecticidal delivery. Imidacloprid (IMI) loaded UiO-66-COOH nanoparticles achieved a loading content of approximately 7 wt%, accompanied by a ζ-potential shift from -6.24 to -18.7 mV and a surface-area reduction from 254.64 to 67.84 m^2^ g^-1^, confirming intrapore filling. After sodium lignosulfonate coating, the resulting IMI@UiO-66@SL formulation displayed improved dispersibility, pH/laccase-responsive release and superior control of *Reticulitermes flaviceps*, with substantially reduced toxicity toward rice seedlings and earthworms (Wu et al., 2025). Similarly, 1,2-distearoyl-sn-glycero-3-phosphoethanolamine-N-[amino(polyethylene glycol)-2000] modified ZIF-8 carriers containing imidacloprid exhibited high aqueous dispersibility, enhanced leaf adhesion and greater intestinal uptake in *Myzus persicae*, achieving stronger field performance with lower chemical input than commercial formulations (Chen et al., 2025a). Chlorantraniliprole delivery has benefited from analogous strategies. Sodium lignosulfonate-modified MIL-101(Fe) (CF@MIL-101-SL) enabled pH- and laccase-triggered release consistent with the alkaline, enzyme-rich midgut of *Spodoptera frugiperda*. The nanocarrier caused marked oxidative stress, vacuolization and epithelial degeneration, while also reshaping the gut microbiota, collectively enhancing chlorantraniliprole lethality by 30-40% relative to the technical (Chen et al., 2023a).

More advanced MOF-based platforms have integrated chemical insecticides with RNA-interference agents. Chitosan-functionalized ZIF-8 capable of co-delivering cycloxaprid and pooled siRNAs provided nuclease protection, synergistic release and rapid internalization in *Diaphorina citri*, achieving over 80% mortality within 48 hours, substantially outperforming either dsRNA or cycloxaprid alone. Gene-silencing efficiencies exceeded 65%, demonstrating the feasibility of combining MOFs with RNAi for next-generation pest-management strategies. Extended-release MOF hybrids further broaden application possibilities (Wang et al., 2025). ZIF-8-PMMA-zein nanocomposites for cypermethrin exhibited sustained release over 32 days and protease-triggered activation within insect digestive tracts. Zn-BDC MOF hybrids co-delivering phoxim and bioavailable Zn^2+^ achieved loading contents of 15-20%, reduced phoxim usage by up to 75% while maintaining full efficacy, and enhanced pest susceptibility through combined chemical toxicity and micronutrient-mediated physiological stress. Schiff-base-modified ZIF-90 nanoparticles loaded with emamectin benzoate (up to 22% loading) demonstrated light- and pH-responsive degradation and produced 1.5-2.0 fold higher mortality against *S. frugiperda* compared with commercial formulations (Ma et al., 2022).

### 3.2 MOF-based fungicides

**Figure 7:**
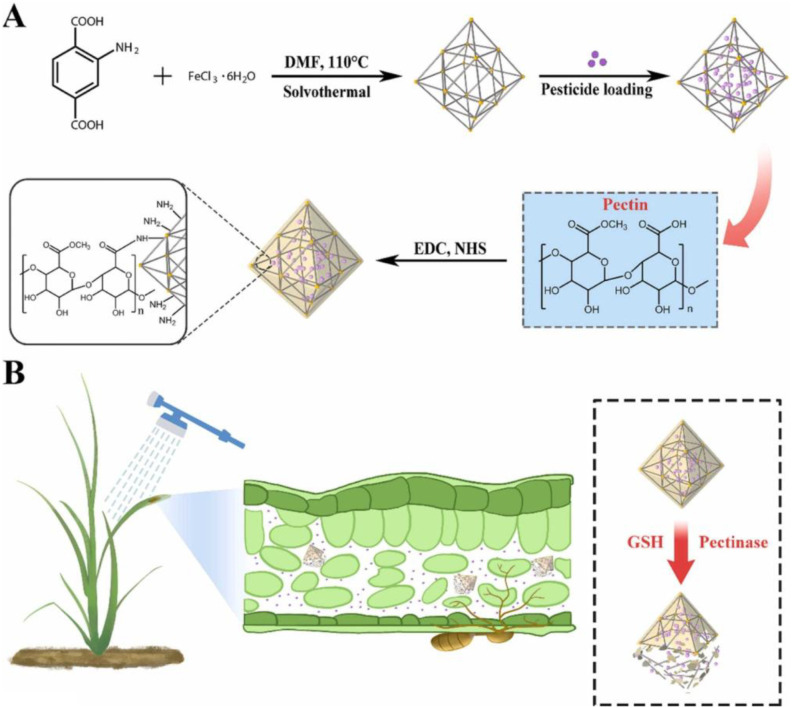
The mechanism for preparation of the PYR@FeMOF-pectin nanoparticles **(A)**. The triggered release mechanism of pyraclostrobin from PYR@FeMOF-pectin nanoparticles **(B)**. Reprinted with permission from ref (Liang et al., 2022a). Copyright 2022, Elsevier.

MOFs have likewise demonstrated considerable potential as nano-scaled carriers for fungicidal delivery, owing to their programmable pore structures, structural robustness, and capacity for pathogen-microenvironment–responsive release. Across diverse MOF architectures, recent studies have reported substantial improvements in fungicide loading efficiency, photostability, pathogen-site activation, and in planta antifungal performance, while simultaneously reducing phytotoxicity and ecological risk. For example, boscalid-loaded UiO-66-NH_2_ coated with a hybrid polysaccharide layer composed of carboxymethylcellulose (CMC) and chitosan quaternary ammonium salt (CQAS). The polymeric gate effectively suppressed premature leakage and enhanced UV tolerance, while the oxalic-acid-rich acidic microenvironment characteristic of *Sclerotinia sclerotiorum* lesions triggered rapid release. Correspondingly, BUCC exhibited a six-fold lower EC_50_ than commercial boscalid water dispersible granules and provided improved foliar retention and excellent biosafety toward earthworms, *Daphnia magna*, and mammalian cells (Yang et al., 2025). Comparable enhancements have been observed for pyraclostrobin-based MOF formulations. PYR@UiO-66@HPC, prepared by coating UiO-66 nanoparticles with hydroxypropyl cellulose, demonstrated dual responsiveness to acidic pH and cellulase secreted by *Rhizoctonia solani*. The nanocarrier enabled greatly accelerated release under infection-associated conditions and delivered significantly higher fungicidal efficacy than pyraclostrobin microcapsule controls, together with 4.6-fold lower toxicity toward *Daphnia magna* and negligible phytotoxicity in rice seedlings (Ma et al., 2023). Similarly, a pectin-gated Fe-MOF system (PYR@FeMOF-pectin) achieved a pyraclostrobin loading of 20.6% and exhibited synergistic pH- and pectinase-responsive release precisely matched to the infection microenvironment of *Magnaporthe oryzae* (**Figure 7**). The formulation protected pyraclostrobin from photolytic degradation, prolonged field persistence, and reduced acute toxicity to zebrafish by approximately eightfold relative to free pyraclostrobin (Liang et al., 2022a).

More advanced MOF-based fungal-control platforms have integrated chemical fungicides with bioactive or regulatory molecules to achieve multifunctional outputs. A ZIF-8-based ternary co-delivery system carrying prochloraz, diethyl aminoethyl hexanoate, and PGase-targeting siRNA enabled pH-triggered disassembly of the ZIF-8 carriers, yielding synchronized chemical and RNAi-mediated inhibition of *Rhizoctonia solani* while simultaneously promoting plant growth. This formulation demonstrated superior disease suppression compared with prochloraz alone and displayed robust biosafety to rice seedlings and soil microorganisms (Huang et al., 2023). Hybrid MOF-inorganic composites have further extended the scope of fungicidal delivery. A pectin-coated Ti-MOF anchored on diatomaceous earth (pectin@prochloraz@Ti-MOF@diatomaceous earth) exhibited an exceptionally high specific surface area (731 m^2^ g^-1^) and a prochloraz loading of 56.5%, together with strong pH/pectinase-triggered release behavior. The formulation achieved 91.5% cumulative release under pathogen-relevant stimuli and provided outstanding control of *Botrytis cinerea*, with field-level efficacy exceeding 95% on tomato fruit and leaves (Bhat et al., 2025).

Hierarchical and hybrid MOF architectures have further broadened the functional scope of nano-fungicide delivery systems by integrating multiple stimuli-responsive elements and leveraging synergistic antimicrobial mechanisms. A representative example is the carboxymethyl cellulose@Eu-ZIF@ZnO tri-layer assembly, which integrates three sequentially engineered components into a unified delivery platform. The outer carboxymethyl cellulose shell functions as a cellulase-degradable gate that restricts premature diffusion. Beneath this layer lies an Eu-doped ZIF interlayer that undergoes controlled degradation in the acidic microenvironments associated with pathogen infection, thereby enabling pH-triggered activation of the MOFs. At the core of the construct, ZnO provides intrinsic antimicrobial activity through the generation of reactive oxygen species. The coordinated behavior of these layers ensures that fungicide release is initiated selectively under infection-relevant biochemical conditions. As a result, the system achieves a cumulative release of 91.5% and reduces the EC_50_ value to approximately one-third of that of free eugenol. The nanostructure also exhibits outstanding rainfastness, retaining 67.9% of its foliar deposition after simulated rainfall, and demonstrates excellent crop safety, underscoring its suitability for field deployment where environmental wash-off and phytotoxicity present significant constraints (Sun et al., 2026).

Bio-derived calcium L-malate and D-tartrate frameworks (UPMOF-1 and UPMOF-2) offer an alternative class of environmentally benign MOF-like carriers for sustained fungicide delivery. These coordination networks exhibited exceptionally high hexaconazole loading (62-63%) and slow, dissolution-governed release, achieving cumulative release rates of 95-98% over more than 500 hours. The sustained release markedly enhanced antifungal efficacy, producing complete inhibition of Ganoderma boninense at only 0.05 μg mL^-1^ and maintaining near-zero disease severity in oil palm seedlings over a 26-week nursery trial. In addition, the controlled release of Ca^2+^ from the frameworks contributed to improved root development, indicating synergistic benefits for both disease suppression and plant health (Ahmad Aljafree et al., 2025).

### 3.3 MOF-based herbicides

MOFs have also emerged as promising platforms for herbicide delivery owing to their highly ordered porosity, tunable coordination environments, and ability to provide sustained, environmentally responsive release. Across diverse MOF architectures, recent studies report substantial improvements in herbicide encapsulation, controlled-release performance, photostability, and weed-control efficacy, together with reduced leaching, soil mobility, and ecotoxicity relative to conventional formulations.

A representative example is the atrazine nanoformulation based on MOF-5. Atrazine-loaded MOF-5 (AT@MOF-5) achieved an adsorption capacity of 26.6 mg g^-1^, accompanied by a decrease in BET surface area from 121.28 to 106.32 m^2^ g^-1^, confirming intrapore occupation. When incorporated into biodegradable polyvinyl alcohol/starch mulch films, atrazine@MOF-5 enabled significantly suppressed soil leaching and a sustained zero-order release profile, maintaining effective herbicidal activity while minimizing off-target contamination (Lee et al., 2022). This demonstrates the potential of integrating MOF reservoirs with degradable matrices to reduce chemical loss in field environments. More advanced MOF architectures have been developed for paraquat delivery. MIL-101(Fe) nanoparticles enabled a high PQ loading of 16.4%, and further encapsulation with a CMC-Ca^2+^ hydrogel produced paraquat @MIL-101(Fe)@CMC-Ca^II^, a multi-stimuli-responsive system triggered by acidic and alkaline pH, glutathione, phosphate, and EDTA. This design effectively suppressed premature leakage, markedly reduced UV-induced paraquat degradation, and prolonged herbicidal performance at reduced dosages. Importantly, the nanoformulation exhibited low toxicity to honeybees and wheat seedlings, highlighting its suitability for safer paraquat delivery (Dong et al., 2023).

**Figure 8:**
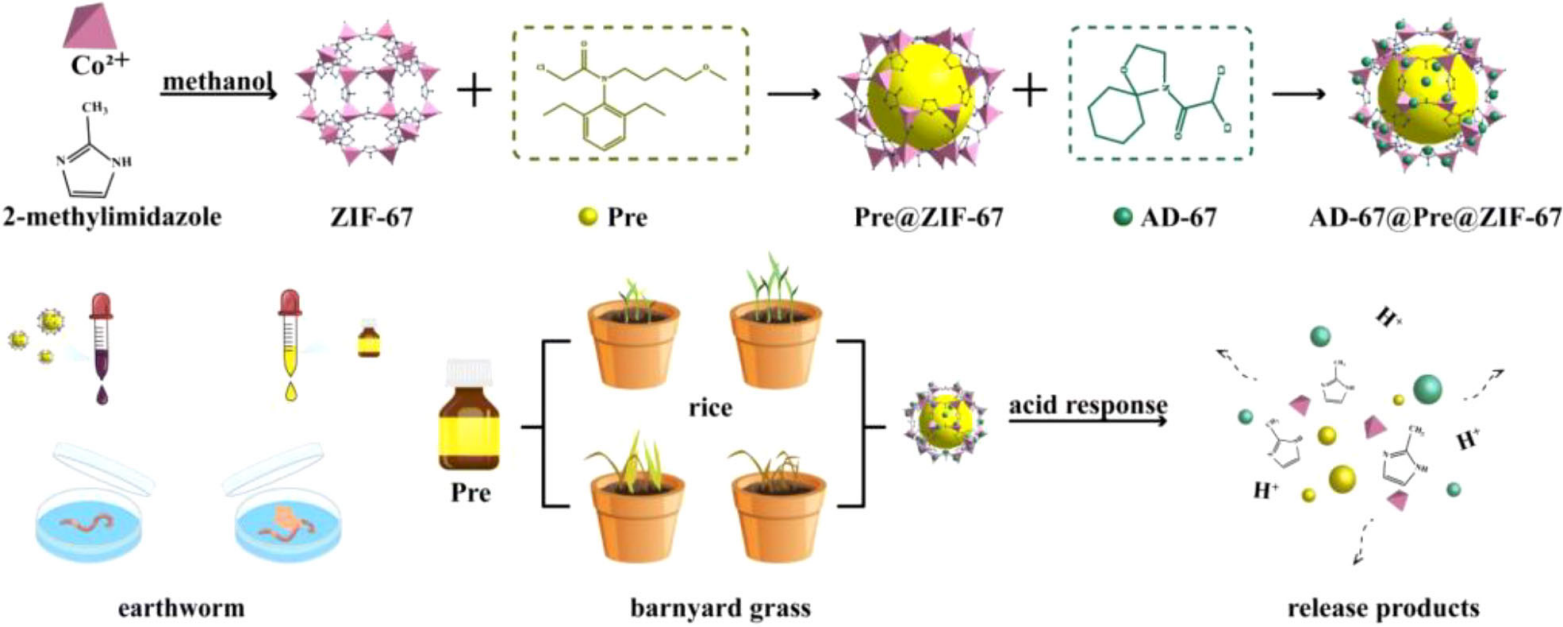
Preparation of AD-67@Pre@ZIF-67 and pH-triggered-release proposal mechanism of Pre and AD-67. Reprinted with permission from ref (Guo et al., 2023). Copyright 2023, Royal Society of Chemistry.

A complementary strategy involves designing “AgroMOFs” in which herbicide molecules themselves serve as coordination ligands. The glyphosine-Cu^2+^ framework GR-MOF-20, which spontaneously transforms into GR-MOF-21 upon hydration, released 74-86% of its ligand content within 24–72 hours. GR-MOF-21 inhibited *Lolium multiflorum* seed germination more strongly (21.7%) than free glyphosine (13.3%) at identical doses while remaining non-phytotoxic to wheat. The Cu^2+^ coordination nodes also imparted intrinsic antibacterial activity against *Escherichia coli* and *Pseudomonas syringae*, illustrating how AgroMOFs can couple controlled herbicide release with auxiliary biological functions (Contreras et al., 2025). Several studies also demonstrate the value of MOF-derived materials in herbicide management. β-cyclodextrin MOF-derived nitrogen- and potassium-doped porous carbon (β-CD MOF-NPC) efficiently adsorbed amide herbicides via π–π interactions, hydrogen bonding, and electrostatic attraction, while simultaneously releasing K^+^ as a plant nutrient. Hydroponic experiments showed that β-CD MOF-NPC alleviated herbicide-induced phytotoxicity and promoted rice seedling growth, revealing the dual functionality of MOF-derived adsorbents in remediation and micronutrient supplementation (Liu et al., 2019). ZIF-based materials have been further engineered as advanced carriers for the controlled release of herbicides (Saklan et al., 2024). Guo et al. reported a pH-responsive ZIF-67 dual-delivery system in which pretilachlor was encapsulated within the framework and the safener 4-(dichloroacetyl)-1-oxa-4-azospiro[4,5]decane (AD-67) was assembled on the particle surface to achieve sequential release under mildly acidic conditions (**Figure 8**). Their results showed rapid AD-67 release (80–90% within 24 h) and sustained zero-order pretilachlor release (≈85% over seven days), accompanied by measurable effects on barnyard-grass control, rice seedling growth parameters, and GST/GSH activity (Guo et al., 2023).

### 3.4 MOF-based plant growth regulators

**Figure 9:**
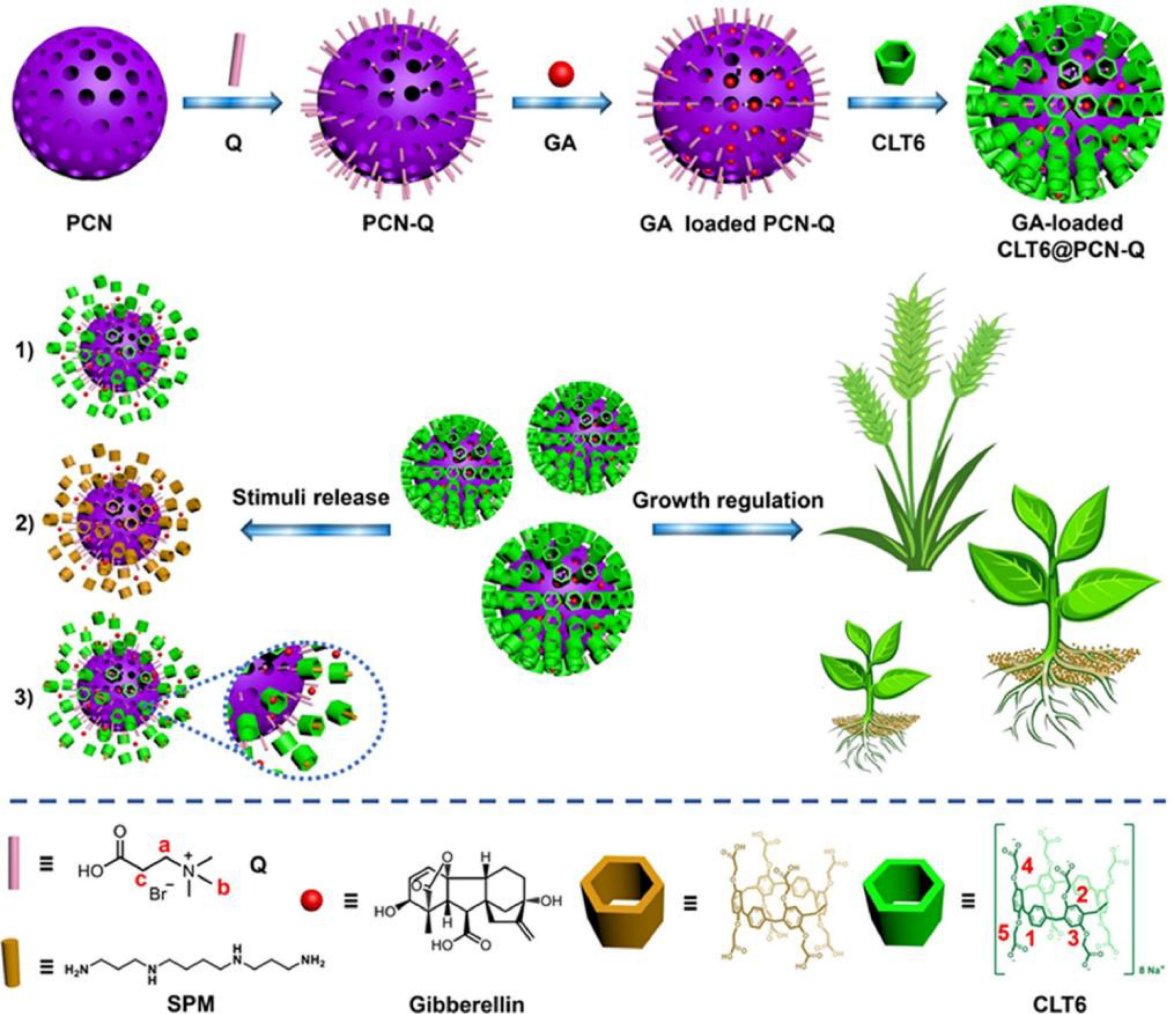
Schematic representation for the fabrication of the multi-stimuli-responsive supramolecular nanoplatform (GA-loaded CLT6@PCN-Q) based on CLT6-capped MOF reservoir, the opening behavior of CLT6 nanovalves under different external stimuli, e.g., 1) temperature, 2) pH, and 3) competitive agent, and the application of GA-loaded CLT6@PCN-Q for the regulation of plant growth. Reprinted with permission from ref (Yang et al., 2021). Copyright 2021, Elsevier.

MOFs have been increasingly explored as platforms for the controlled delivery and regulation of plant growth regulators (PGRs), enabled by their ordered porosity, tunable host–guest interactions, and responsiveness to environmental stimuli. A representative example is the pH-responsive ABA@MIL-100(Fe) system in which abscisic acid (ABA) is encapsulated within the MIL-100(Fe) framework to achieve drought-related microenvironment-triggered release. Wu et al. reported that ABA@MIL-100(Fe) reached a loading rate of 22.2% and released 70.5% of ABA at pH 7.2 within 3 h, while encapsulation strongly reduced ABA photodegradation and induced ABA-dependent physiological responses in cotton seedlings under drought stress (Wu et al., 2025). Beyond ABA delivery, supramolecular MOF-based platforms have been developed for other PGRs. Zhang et al. designed a photo-responsive polysaccharide-MOF hydrogel for gibberellin (GA) and naphthaleneacetic acid (NAA) delivery, with approximately 70% of NAA and 63% of a GA model compound released within 8 h under simulated sunlight, and these systems produced measurable effects on early seedling growth in Chinese cabbage and alfalfa (Zhang et al., 2023). In addition, Yang et al. constructed a multi-stimuli-responsive GA delivery system, CLT6@PCN-Q, in which PCN-type nanoscale MOFs were modified with quaternary ammonium stalks and subsequently capped with carboxylated leaning-tower[6]arene (CLT6) nanovalves through host–guest interactions (**Figure 9**). The resulting supramolecular nanoplatform (approximately 101 nm in diameter) exhibited controlled release in response to pH, temperature, and competitive agents such as spermine, and GA-loaded CLT6@PCN-Q effectively promoted seed germination and stem elongation in Chinese cabbage and wheat (Yang et al., 2021).

MOFs have also been explored in depth for regulating gaseous plant growth regulators, particularly ethylene, which plays a key role in the ripening of climacteric fruits. Porous CuTPA MOFs have been applied as solid-state ethylene storage and release carriers, taking advantage of their high surface area and affinity for small gaseous molecules. In this system, ethylene molecules diffuse into the microporous CuTPA framework and are temporarily stabilized through weak coordination and van der Waals interactions. A 50 mg sample of CuTPA absorbed 654 μL/L ethylene during loading, and desorption studies revealed that 95.8% of the stored ethylene was released within 180 min once the MOF was exposed to ambient conditions. The release profile showed an initial rapid desorption phase followed by a slower diffusion-controlled stage, consistent with gas release from microporous crystalline materials. When applied to bananas and avocados, the released ethylene accelerated typical ripening-related physiological changes. Bananas showed a marked reduction in peel firmness and rapid color transition from green to yellow within 24 h, while avocados softened significantly and exhibited characteristic skin darkening, demonstrating that MOF-released ethylene can effectively mimic the action of conventionally applied gaseous ethylene but in a more controlled and safer solid-state format (Zhang et al., 2016). Related work has expanded MOF-based ethylene delivery into polymeric matrices to create practical postharvest packaging. For example, Al-MOF particles have been embedded into alginate-based films that remain inert during storage, but release ethylene once exposed to citrate ions. This citrate-triggered release is achieved through ligand displacement at the Al–carboxylate coordination nodes, which destabilizes the adsorbed ethylene and facilitates controlled desorption. Such films allow fruits to be transported without active ethylene exposure and then ripened on demand by a simple citrate rinse. This approach avoids the safety and logistical risks associated with compressed ethylene cylinders and provides uniform ripening throughout the packaged batch (Guan et al., 2019). Beyond direct ethylene storage and release, MOFs have also been integrated into active packaging to influence ethylene-mediated physiological processes during vegetable storage. Melatonin-loaded UiO-66, for instance, incorporated into sodium alginate films, gradually released melatonin—a gaseous PGR-related antioxidant regulator—during storage. In spinach preservation studies, these films modulated oxidative metabolism by influencing SOD, POD, and CAT enzyme activity, reduced yellowing, maintained leaf turgidity, and extended shelf life. Although melatonin does not act as an ethylene analog, the MOF-based film helped counteract ethylene-associated senescence symptoms, illustrating another functional route by which MOFs can indirectly regulate hormone-related postharvest physiology (Wang et al., 2024b).

### 3.5 Integrated comparative analysis of MOF-based pesticide delivery

Across insecticidal, fungicidal, herbicidal, and plant growth regulator applications, MOFs consistently function as multifunctional delivery platforms that combine high loading capacity, protection of active ingredients, and environmentally responsive release. However, the specific design priorities and release logics diverge substantially depending on the biological target and application context. For insecticidal delivery, MOF systems are predominantly engineered for rapid activation within insect digestive environments. Acidic pH, digestive enzymes such as amylase or protease, and intestinal redox conditions are frequently exploited as triggers to induce fast framework degradation or gate opening. Consequently, insecticidal MOFs emphasize particle-size control, surface charge modulation, and enhanced intestinal uptake to achieve acute toxicity and rapid mortality. Hybrid strategies integrating chemical insecticides with RNA-interference agents further highlight the need for efficient cellular internalization and synchronized release in insect systems. In contrast, fungicidal MOF platforms are primarily designed around recognition of pathogen-associated microenvironments at infection sites. Acidic lesion zones, cell-wall-degrading enzymes (e.g., cellulase, pectinase), and pathogen-secreted metabolites serve as dominant stimuli for controlled fungicide release. Compared with insecticides, fungicidal systems place greater emphasis on prolonged field persistence, rainfastness, and compatibility with plant tissues, reflecting the chronic and spatially localized nature of fungal infections. Hierarchical and hybrid MOF architectures integrating polysaccharide gates, inorganic antimicrobial components, or bioactive regulators exemplify this infection-responsive and durability-oriented design philosophy. For herbicidal applications, the central objective shifts from rapid biological activation to sustained environmental control. MOF-based herbicide formulations are therefore optimized to suppress leaching, volatilization, and off-target diffusion in soil–plant systems. Sustained or near zero-order release profiles are commonly pursued, often through integration with hydrogels, polymer films, or soil matrices. In some cases, herbicide molecules themselves act as organic linkers to form so-called AgroMOFs, blurring the distinction between carrier and active ingredient and enabling simultaneous delivery of herbicidal and auxiliary functions such as micronutrient release or antimicrobial activity. MOF-based plant growth regulator systems exhibit yet another distinct design logic, characterized by precise dose control and temporal regulation rather than toxicity-driven performance. These systems typically operate at lower active-ingredient loadings and rely on mild environmental triggers such as light, temperature, or subtle pH variations. Gas-regulating MOFs for ethylene storage and release further expand MOF applications beyond conventional liquid delivery, enabling solid-state hormone management and postharvest regulation. In this context, MOFs function not only as carriers but also as regulators of plant physiological processes. These comparisons reveal that while high surface area, tunable porosity, and stimuli responsiveness constitute universal advantages of MOFs, their successful agricultural deployment depends on application-specific design rather than framework generalization. Rational selection of MOF composition, pore architecture, surface chemistry, and degradation behavior must therefore be guided by the biological target, exposure pathway, and desired temporal release profile.

## 4 Future perspectives and outlook

Despite the promising performance of MOF-based delivery systems across insecticidal, fungicidal, herbicidal, and plant growth regulator applications, their large-scale agricultural deployment is still constrained by several shared challenges related to sustainability, environmental safety, economic feasibility, and regulatory acceptance. First, the sustainability and cost of MOF synthesis represent a fundamental bottleneck. Many MOFs are synthesized using organic solvents that are potentially toxic, flammable, and environmentally persistent, raising concerns regarding human health, volatile organic compound emissions, and ecosystem contamination. These concerns are particularly pronounced in agricultural contexts, where large production volumes and open-field application may amplify both environmental exposure and manufacturing costs. Consequently, the development of low-cost metal sources, bio-derived or commodity ligands, and green synthesis strategies—such as aqueous, solvent-free, or mechanochemical methods—is essential to improve both sustainability and economic competitiveness. Second, the environmental fate of MOFs remains insufficiently understood and is highly application dependent. Although most reported MOFs exhibit low acute toxicity, their long-term behavior in soil–plant–water systems have not been systematically evaluated. Emerging evidence suggests that Fe- and Zn-based MOFs, such as MIL-101(Fe) and ZIF-8, can gradually degrade under soil-relevant conditions, releasing metal ions at levels comparable to those of conventional micronutrient fertilizers, with limited short-term toxicity toward non-target organisms. In contrast, highly stable frameworks, including Zr-based UiO-type MOFs, may persist for extended periods, raising concerns regarding environmental accumulation and transformation products. Importantly, different pesticide application scenarios further modulate environmental risk profiles. In insecticidal and fungicidal systems, MOFs are often designed to degrade rapidly in biologically active microenvironments, leading to localized release of active ingredients and framework components. Conversely, herbicidal formulations frequently emphasize sustained or near zero-order release, increasing the likelihood of prolonged environmental residence. Plant growth regulator systems, particularly those used in postharvest treatments or packaging applications, may introduce additional exposure pathways through food-contact materials. These distinctions underscore the necessity of application-specific ecological risk assessments rather than generalized toxicity evaluations. Third, economic feasibility and farmer adoption are decisive for practical implementation. Beyond performance and safety, MOF-based formulations must demonstrate clear cost–benefit advantages relative to conventional pesticides. Factors such as synthesis scalability, formulation stability, ease of handling, and compatibility with existing agricultural equipment will strongly influence farmer acceptance and adoption. Finally, regulatory uncertainty constitutes a shared barrier across all application categories. Current regulatory frameworks are largely designed for conventional pesticide formulations and do not adequately address nanoscale features such as framework degradation kinetics, stimulus-responsive release behavior, or transformation products. To overcome these limitations, future research should integrate life-cycle assessment, long-term environmental fate studies, and unified evaluation criteria into MOF-based pesticide development. Establishing standardized protocols for characterizing release behavior, persistence, and ecological safety will not only facilitate regulatory approval but also enable more rational, application-driven design of MOF-based agricultural technologies.

## 5 Conclusion

In summary, MOFs represent a versatile and powerful class of materials for advancing agrochemical delivery in modern agriculture. Owing to their tunable porosity, large specific surface area, and modular structure, MOFs enable high loading capacity, protection of active ingredients, and precisely controlled release, thereby addressing critical limitations of conventional agrochemical formulations such as low utilization efficiency, rapid degradation, and environmental contamination. Beyond their function as delivery carriers, MOFs offer multifunctional advantages that are highly relevant to sustainable crop production. Stimuli-responsive MOF systems can release agrochemicals in response to microenvironmental changes induced by pests or pathogens, improving spatiotemporal targeting and reducing unnecessary chemical input. In addition, certain MOFs can act as sources of beneficial macro- and micronutrients, integrating crop protection with plant nutrition and stress regulation. Despite these promising attributes, several challenges must be overcome before MOF-based agrochemical systems can be widely implemented. The transport, distribution, and transformation of MOFs within soil–plant systems remain insufficiently understood and require systematic toxicological and ecological evaluation. Furthermore, future research should prioritize enhancing pesticide loading efficiency, reducing synthesis cost, and developing green, scalable fabrication strategies to ensure economic feasibility and regulatory acceptance. Thus, MOF-based nanocarriers hold significant potential to reduce agrochemical consumption, enhance pest and disease control efficiency, and promote environmentally responsible agricultural practices. With continued interdisciplinary efforts spanning materials science, plant protection, and agricultural engineering, MOF-enabled delivery platforms are expected to contribute meaningfully to the development of smart, efficient, and sustainable modern agriculture.
